# Conditional knockdown of integrin beta-3 reveals its involvement in osteolytic and soft tissue lesions of breast cancer skeletal metastasis

**DOI:** 10.1007/s00432-020-03428-y

**Published:** 2020-10-20

**Authors:** Marineta Kovacheva, Michael Zepp, Stefan Berger, Martin R. Berger

**Affiliations:** 1 Toxicology and Chemotherapy Unit, German Cancer Research Center (DKFZ), 69120 Heidelberg, Germany; 2 Department of Molecular Biology, Central Institute of Mental Health, 68159 Mannheim, Germany

**Keywords:** Conditional ITGB3 knockdown, Integrins, Doxycycline, Breast cancer, Skeletal metastasis

## Abstract

Integrin β3 (ITGB3) is probably related to skeletal metastasis, which is the most frequent complication in breast cancer progression. We aimed to define its role and suitability as target for anti-metastatic therapy. We generated two MDA-MB-231 cell clones with conditional miRNA-mediated ITGB3 knockdown for analyzing the resulting effects in vitro regarding mRNA expression, proliferation and migration, as well the impact on skeletal metastasis in a nude rat model. Furthermore, ITGB3 levels were analyzed in exosomes from plasma of rats with skeletal metastases, and from MDA-MB-231 cells incubated with these vesicles, as well as from exosomes secreted by cells with conditional ITGB3 knockdown. This inhibition of ITGB3 expression decreased cellular proliferation and more distinctly inhibited cellular migration. Reduction and even complete remissions of respective soft tissue and osteolytic lesions were detected after ITGB3 knockdown in vivo. Furthermore, ITGB3 levels were increased in exosomes isolated from plasma of rats harboring MDA-MB-231 lesions as well as in respective cells incubated with these vesicles in vitro. ITGB3 was distinctly decreased in exosomes from cells with ITGB3 knockdown. The observed in vitro and in vivo anti-ITGB3 effects can be explained by downregulation of specific genes, which have roles in angiogenesis (*NPTN, RRM2*), tumor growth (*NPTN*), energy metabolism (*ISCA1*), cytokinesis (*SEPT11*), migration (*RRM2, STX6*), cell proliferation, invasiveness, senescence, tumorigenesis (*RRM2*) and vesicle trafficking (*SEPT11, STX6*). ITGB3 has a role in breast cancer skeletal metastasis via gene expression modulation, as mirrored for ITGB3 in exosomes, thus it could serve as target for anti-metastatic therapy.

## Introduction

Integrins are a family of transmembrane proteins, which integrate the extracellular matrix processes and the intracellular compartment activities (Raab-Westphal et al. 2017). To date, 24 integrin heterodimers have been identified, which are formed by combining 18 α- and 8 β-subunits (Hamidi et al. 2016). Multiple activation states, splice variants and heterogeneous glycosylation give rise to different functional and tissue specificities (Raab-Westphal et al. 2017). Integrins are bi-directional signaling molecules with different conformation states, i.e., they have a bent (closed) or fully extended (open) form. In contrast to the first form, the second one is active and with high affinity to specific extracellular matrix (ECM) ligands. Following ligand binding to the active integrins, mostly via the short amino-acid sequence arginine-glycine-aspartic acid (RGD) (Raab-Westphal et al. 2017), downstream signaling and cellular responses are provoked (‘outside-in’ signaling) (Hamidi and Ivaska 2018). Integrins can respond also to inside signals, for example, active talin can bind to the β-integrin subunit, which causes the receptor to turn into its extended open conformation (‘inside-out’ signaling). Moreover, active integrins and those along with ECM ligands or receptor tyrosine kinases are localized within endosomes, where they can trigger ‘inside-in’ signaling (Hamidi and Ivaska 2018). Both α and β subunits are binding to the ligand, but only β chains determine the cytoskeletal interactions (Pan et al. 2016).

Integrin expression varies between normal and tumor tissues (Desgrosellier and Cheresh 2010). β1 and β3 integrins are expressed on malignant breast cancer cells and are involved in their adhesion (Das et al. 2018). It is known that integrin αvβ3 has low or undetectable levels of expression in most adult epithelia and resting endothelial cells in non-diseased tissues, whereas in breast and prostate cancers, and in activated tumor endothelial cells it is highly expressed (Desgrosellier and Cheresh 2010; Bauerle et al. 2011). Integrin αvβ3 interacts with the integrin-binding tripeptide (RGD) of secreted proteins from the SIBLING family, as osteopontin (OPN) and bone sialoprotein (BSP) (Weilbaecher et al. 2011). In addition, active matrix metalloproteinase 2 (MMP2) can bind to αvβ3, and then is localized on the cellular surface for executing its proteolytic activity at the leading edge of the invasive cell (Karadag et al. 2004). Integrin αvβ3 is related to high rates of bone metastasis, tumor-associated osteolysis and colonization to the bone (Weilbaecher et al. 2011). Bone metastasis is the most frequent complication in breast cancer, which is occurring in 70% of the affected patients (Roodman 2004). This complication has devastating consequences and increases the risk of skeletal related events like severe pain, pathologic fractures, life-threatening hypercalcemia, alteration of hematopoiesis by bone marrow infiltration, spinal cord or nerve roots´ compression, which may have decisive impact on the quality of life, morbidity and mortality of cancer patients (Roodman 2004; Uccello et al. 2011). The lack of novel treatment led to the urgent necessity of deciphering the mechanism of the metastatic process and of finding a valuable target for anti-metastatic therapy.

Based on this pathophysiology, integrin β3 (ITGB3) emerged as a potential target for therapy of breast cancer skeletal metastasis. Several approaches for ITGB3 inhibition were investigated and gave promising results. Targeting of ITGB3 mRNA by siRNA significantly inhibited the migration of breast cancer cells in vitro (Reufsteck et al. 2012). The cyclic pentapeptide Cilengitide having the sequence c(Arg-Gly-Asp-D-Phe-N-Me-Val) exerts high affinity for αvβ3 and αvβ5 and inhibits their cellular processes. This peptide partially suppressed bone resorption and soft tissue tumor growth in osseous lesions in experimental breast cancer bone metastases (Bauerle et al. 2011). In the current approach, we established a model for better understanding of the role of ITGB3 in breast cancer skeletal metastasis, avoiding the disadvantages of permanent knockout (long-term adaptation to the protein deficiency, which could be basis for potential survival strategies of the tumor cells) and that of a transient knockdown (only short-term protein inhibition). We generated MDA-MB-231 breast cancer cells with conditional doxycycline-dependent miRNA-mediated integrin β3 knockdown. This model allowed monitoring the effects of ITGB3 inhibition at definite point in time on cellular properties and gene modulation (in vitro) and on breast cancer skeletal metastasis (in vivo). Recently, integrins were found to be present on the surface of exosomes, which are small membrane vesicles, shed by normal and cancerous cells that contain RNA and proteins (Hamidi and Ivaska 2018). The tumor-derived exosomes are supposed to play a role for the preparation of a pre-metastatic niche by fusing with target cells in a tissue-specific fashion (Hoshino et al. 2015) and by triggering the expression of specific ECM components to support cancer cell survival. Furthermore it was shown that some integrins guide their exosomes to particular tissue sites, as, e.g., exosomes containing integrins α6β1 or α6β4 are guided to the lungs and αvβ5 integrin positive exosomes to the liver for preparing pre-metastatic niches (Hamidi and Ivaska 2018). Therefore, we analyzed the presence of ITGB3 also in exosomes produced by MDA-MB-231 cell clones with conditional integrin β3 knockdown, as well as in exosomes isolated from plasma of rats with breast cancer skeletal metastasis.

## Materials and methods

### Generation and cultivation of breast cancer cell clones

Two MDA-MB-231 breast cancer cell clones with conditional ITGB3 knockdown (I3 and I5) were generated by a previously described procedure (Kovacheva et al. 2014). The miRNA integrated into the genome of these cell clones includes the respective DNA sequence of previously tested siRNA (siRNA targeting ITGB3 mRNA: GAAAAUCCGUUCUAAAGUA) (Reufsteck et al. 2012). They were cultivated in RPMI 1640 medium (Invitrogen, Karlsruhe, Germany), supplemented with 10% fetal calf serum (FCS), 100 ng/ml doxycycline, 2 mM l-glutamine, 100 U/ml penicillin, and 100 µg/ml streptomycin (Invitrogen, Karlsruhe, Germany) in cell culture flasks (TPP, Trasadingen, Switzerland) at 37 °C and 5% CO_2_ in a humidified incubator. The human breast adenocarcinoma cell line MDA-MB-231 was obtained from ATCC (Manassas, VA, USA) and the parent cell clone was authenticated by Multiplex Cell Authentication (Multiplexion, Heidelberg, Germany) as described (Castro et al. 2013). The single nucleotide polymorphism (SNP) profiles matched known profiles.

### Flow cytometry analysis

The generated cell clones were cultivated in small culture flasks (25 cm^2^, TPP, Trasadingen, Switzerland) in medium with or without doxycycline for 6 days. Then, the cells were prepared for flow cytometry analysis as described previously (Kovacheva et al. 2014,2019). The analyses were done to ensure the purity (> 98%) of successfully transfected cell clones and to confirm the activation of the mCherry expression (respectively the activation of the Tet-Off system) in these cells after cultivating them in medium without doxycycline.

### Quantitative polymerase chain reaction (qPCR)

The cell clones I3 and I5 were cultivated in medium with or without doxycycline for 6 days, then harvested and their total RNA was isolated by an RNeasy mini-kit (Qiagen, Hilden, Germany), according to the respective manufacturer’s protocol. The corresponding cDNA was synthesized from the isolated RNA in a reaction mixture of 1000–1500 ng RNA, buffer RT (1x), dNTPs (0.5 mM), oligo-dT-primers (1 μM), RNase inhibitor (20 units) and reverse transcriptase (8 units), and for an incubation time of 1 h at 37 °C. The Omniscript RT Kit was used for cDNA synthesis according to the manufacturer’s protocol (Qiagen, Germany). The subsequent quantitative PCR was performed with the LightCycler 480 Real-Time PCR system and the human Universal Probe Library kit (Roche, Mannheim, Germany) according to the manufacturer’s protocol. The procedure was performed as described previously (Kovacheva et al. 2019).

### Western blot

Immunoblotting was done as described in (Yosifov et al. 2007), but a proteinase inhibitor cocktail (Roche, 25x) was used and additional dithiothreitol was not included to the sample mix and loading dye. Aliquots of the cell lysates were further analyzed as described before (Kovacheva et al. 2014). Specific antibodies detected the following proteins: integrin β3 (sc-46655) and β-actin (sc1615) (Santa Cruz, Heidelberg, Germany). Beta-actin served as internal loading control. The respective horseradish-peroxidase-conjugated secondary antibodies were goat anti-mouse (sc 2055) and donkey anti-goat (sc2020) (Santa Cruz, Heidelberg, Germany).

### Proliferation and migration assays

The 3-(4,5-dimethylthiazol-2-yl)-2,5-diphenyltetrazolium bromide (MTT) assay was used to assess the effect of ITGB3 knockdown on the proliferation of breast cancer cells. Briefly, 2–3 × 10^3^ cells/well were seeded into six-well plates and cultivated for 6 days in media with or without doxycycline. Then, the assay was performed as described previously (Kovacheva et al. 2019).

The migration assay was performed after cultivating the control and specific cell clones (I3 and I5) in medium with or without doxycycline for 6 days. Then, the cells were harvested, re-suspended in medium without FCS and 1 × 10^5^ cells were seeded into hanging cell culture inserts with 8 µm pore size membranes (Millicell, Millipore, Switzerland) (upper compartment). These inserts were transferred into previously prepared wells of 24-well plates, containing medium with 10% FCS with or without doxycycline (lower compartment). The cells were allowed to migrate into the lower compartment for 2–3 days, then the migrated cells were detected by Cell Titer Blue Reagent (Promega, Mannheim, Germany), according to the manufacturer’s protocol. The fluorescence was measured by a fluorescence reader (Synergy 2, Biotek, Germany) with excitation filter: 560/15 and emission filter: 590/20.

### Animal experiments—animals, tumor cell inoculation, setup of the experiments, tumor size determination

Four to six weeks old male nude rats (RNU strain, Charles River, Germany) were kept as described previously (Kovacheva et al. 2019). The responsible governmental animal ethics committee (Regierungspräsidium Karlsruhe, Germany) approved all animal experiments. The experiments started after 1 week used as adaptation period.

Three days prior to tumor cell inoculation, the experimental rats received doxycycline via the drinking water (2 µg/ml), which was supplemented with 240 mg sodium cyclamate and 24 mg saccharin-sodium per liter to hide the bitter taste of doxycycline. Then, cell suspensions of the I5 cell clone were prepared and injected into the saphenous artery of four rats as described previously (Kovacheva et al. 2014; Zepp et al. 2011). Two weeks after tumor cell inoculation, the doxycycline intake was discontinued and concomitantly the expression of specific miRNA targeting ITGB3 was stimulated. The respective effect on tumor size was observed by bioluminescence imaging (BLI), magnetic resonance imaging (MRI) and volume computed tomography (VCT) as described before (Zepp et al. 2011).

### Isolation and lysis of exosomes

Exosomes were isolated from plasma of healthy rats and of rats harboring MDA-MB-231-induced skeletal metastasis by ExoEasy Maxi Kit (Qiagen) and according to the manufacturer’s protocol. The tumor cells inducing skeletal metastases grew for 2–3 weeks until they reached a volume, giving rise to a light intensity of 10^6^–10^7^ p/s/cm^2^/sr. In addition, parent and I5 clone cells were cultivated for 6 days in medium containing vesicle-free FCS with or without doxycycline. Then, the exosomes contained in the cultivating media were isolated by the same procedure. Aliquots of these vesicles (60 μl) were lysed by 5 × RIPA buffer supplied with protease inhibitors (15 μl) on a rotator for 30 min at 4 °C. Then, the lysates were centrifuged at 4 °C for 20 min at 14000 rpm. The proteins in the supernatant of the lysates were analyzed by Western blot.

### Microarray analysis

Microarray analysis was performed as described (Kovacheva et al. 2019; Georges et al. 2011). As test for significance, the Student’s *t*-test was used for the bead expression values of the two groups of interest. The average expression value was given as mean of the measured bead expression values and the corresponding standard deviation. Modulations in gene expression were considered significant if the *p* value corrected by the Benjamini–Hochberg procedure was less than 0.01.

### Statistics

The multiple measurements of in vitro functional tests were given as mean with corresponding standard deviation. The independent appearance of the investigated in vitro functional properties was tested by two-way analysis of variance (ANOVA) and Bonferroni post hoc test. For the in vivo experiments, the unpaired *t*-test was used, which was corrected for multiple comparisons using the Holm-Sidak method, as provided by the program GraphPad Prism 6. *p* values lower than 0.05 were considered significant.

## Results

### Generation and characterization of MDA-MB-231 cell clones

**Fig. 1:**
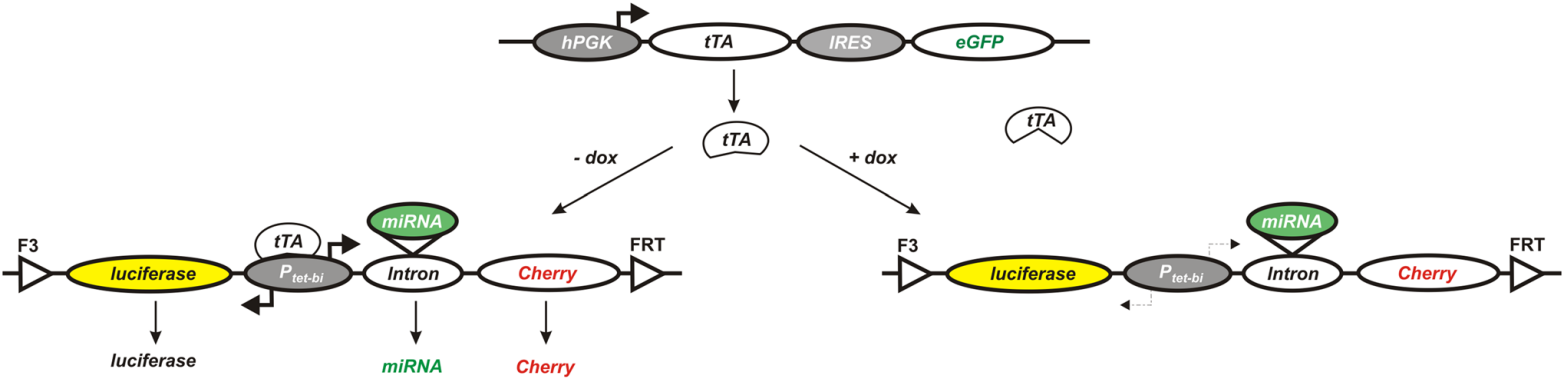
Gene cassettes integrated into the genome of MDA-MB-231 breast cancer cells. Scheme of two cassettes, which were transduced into MDA-MB-231 breast cancer cells. The upper cassette (transduced initially) contains: hPGK (human phosphoglycerate kinase promoter); tTA (tetracycline-controlled transactivator); IRES (internal ribosome entry site); eGFP (enhanced green fluorescent protein). The lower cassettes contains luciferase (firefly luciferase); P_tet-bi_ (bidirectional tet-regulated promoter); Cherry (red fluorescent protein mCherry); FRT and F3 (wild-type and mutant Flp—recombinase target sites); miRNA—artificial microRNA targeting specifically ITGB3 mRNA. The left bottom part illustrates the conditions in absence of doxycycline: tTA binds and activates the bidirectional promoter P_tet-bi_, which leads to expression of firefly luciferase, red fluorescent protein mCherry and of the miRNA targeting ITGB3 mRNA. The right bottom part shows the consequences of doxycycline presence: tTA cannot bind to its respective promoter, it remains inactive and the expression of the subsequent genes is switched off

MDA-MB-231 cells were basis to create a parent cell clone by successively transducing two gene cassettes. The first cassette comprised genes for constitutive expression of tetracycline-controlled transactivator (tTA) and eGFP. The second cassette contained genes for expression of firefly luciferase and red fluorescent protein mStrawberry. This gene cassette was flanked by the wild-type and mutant Flp-recombinase target sites FRT and F3. These two recombinase target sites allowed exchanging this control cassette for a gene cassette containing firefly luciferase, red fluorescent protein mCherry and a highly specific and efficient miRNA targeting ITGB3 mRNA (Fig. 1). This procedure (Kovacheva et al. 2014) was used to generate the specific cell clones I3 and I5 with conditional ITGB3 knockdown. In absence of doxycycline, tTA binds to the bidirectional Ptet promoter and activates the simultaneous expression of firefly luciferase, red fluorescent protein mCherry and of the miRNA targeting ITGB3 mRNA. However, in the presence of doxycycline, tTA cannot bind to the respective promoter for its conformational change caused by the binding of doxycycline. Therefore, the expression of the Ptet regulated genes is switched off.

**Fig. 2:**
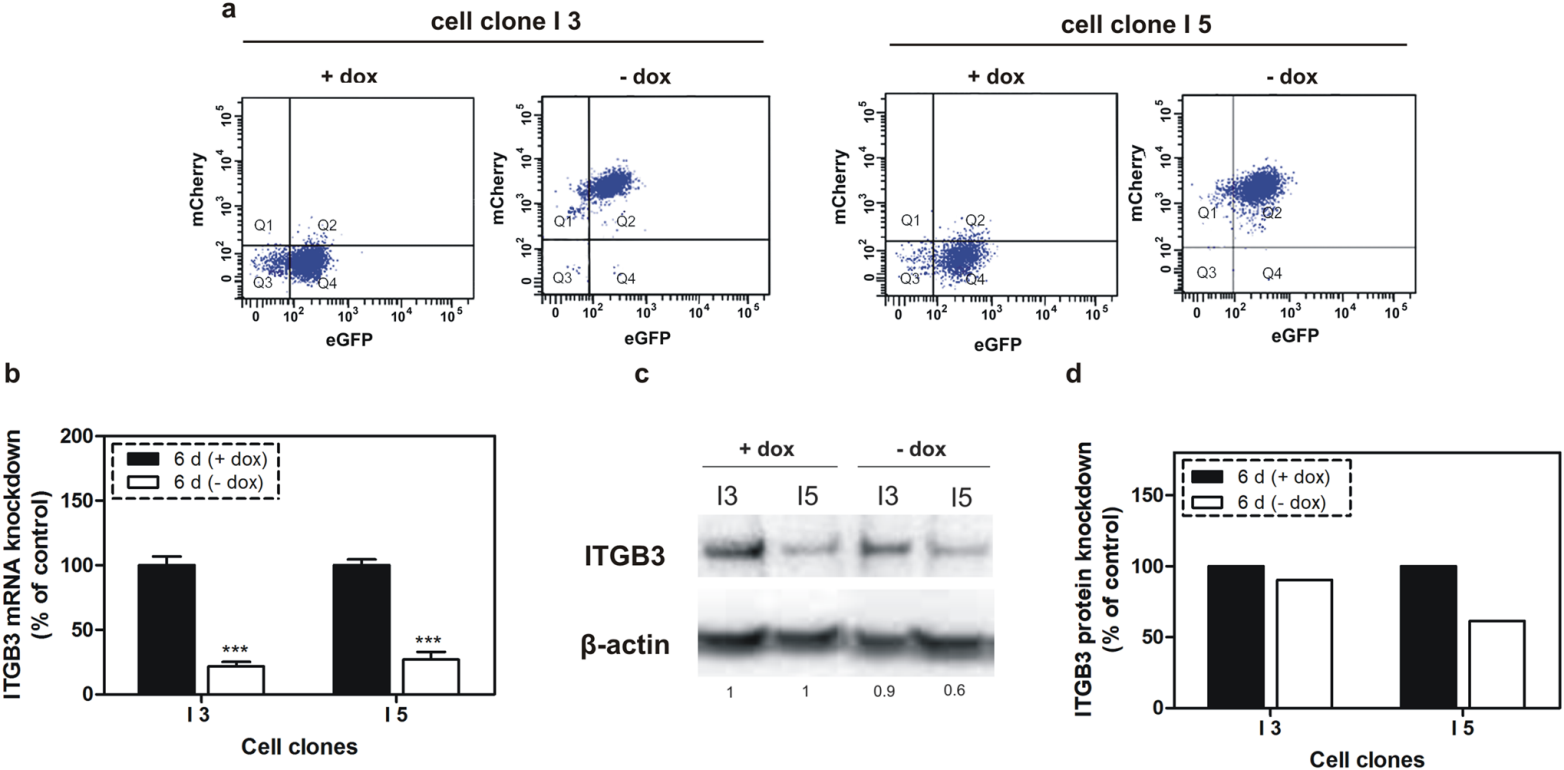
Regulation of the red fluorescent protein mCherry by doxycycline and ITGB3 mRNA and protein levels following conditional knockdown. **a** Flow cytometry analysis of I3 and I5 cell clones after cultivating them for 6 days in media with or without doxycycline. mCherry expression was stimulated after removal of the doxycycline from the media of the cells. **b** qPCR analysis of the mRNA levels of ITGB3 in cell clones I3 and I5, cultivated for 6 days in media with or without doxycycline. Integrin β3 mRNA was significantly inhibited upon stimulated expression of specific miRNA. **c** Western blot analysis of ITGB3 in I3 and I5 cells after cultivating them in media with or without doxycycline for 6 days. **d** Quantitation of the western blot analysis

**Fig. 3:**
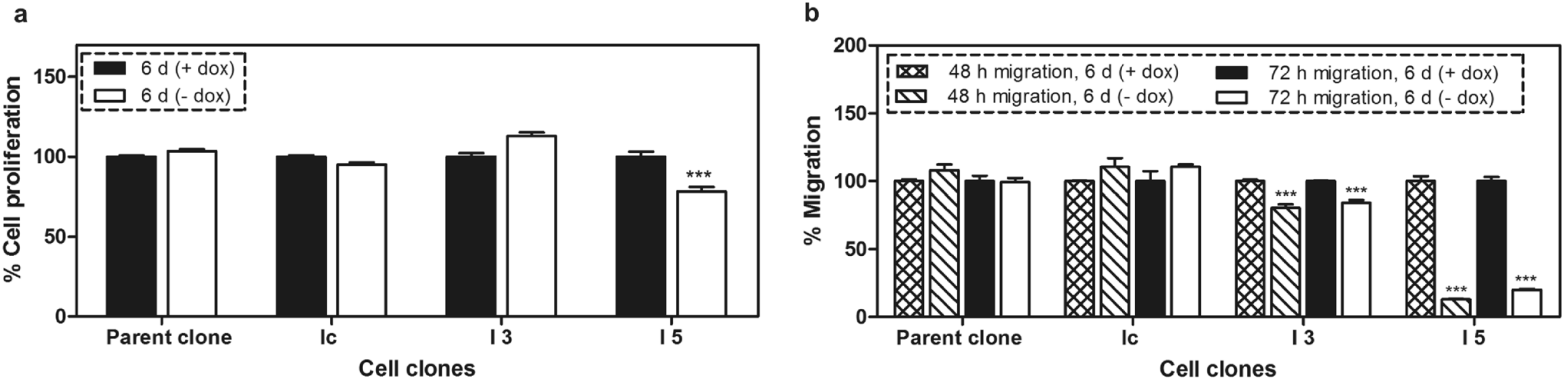
Cellular functions of breast cancer cell clones upon 6 days of conditional miRNA mediated ITGB3 knockdown **a** Cellular proliferation determined by MTT assay; **b** cellular migration; these assays were run with two controls: the parent cell clone and an additional control clone (Ic), which contains the gene cassette without miRNA sequence. The conditional ITGB3 knockdown provoked slight, but significant inhibition of proliferation in I5 cells only, whereas migration was decreased in both cell clones I3 and I5, yet more pronounced in I5 cells. ***p < 0.001 versus controls

When individual clones had reached sufficient cell numbers, they were characterized and the efficiency of the gene regulation by doxycycline was analyzed by flow cytometry, qPCR and western blot after cultivating the created cell clones in media with or without doxycycline. As shown by the flow cytometry analysis, mCherry reporter gene expression was increased by two orders of magnitude in these cell clones in response to removal of doxycycline from cell media for 6 days (Fig. 2a). Under these conditions, the stimulated expression of specific miRNA targeting ITGB3 caused inhibition of ITGB3 in I3 and I5 cell clones by 78% and 73% at mRNA and by 10% and 40% at protein levels, respectively (Fig. 2b–d). The observed 30% difference in protein knockdown between both cell clones I3 and I5 is related to protein translation following a successful inhibition of transcription. The difference is possibly due to the propagation of two independent cell clones from successfully transfected MDA-MB-231 cells, as high levels of ITGB3 may favor the proliferation of these cells during the selection process. After removal of doxycycline from the cultivation media for 6 days, the cell proliferation decreased only in I5 cells (by 22%), whereas the migration was inhibited in both cell clones, but more pronounced in I5 (87%) than in I3 cells (20%), corresponding to an effect related to the ITGB3 concentration (Fig. 3). In parallel, proliferation and migration were not significantly changed in the control cell clones (parent and Ic clones), which have similar gene cassettes, but without miRNA targeting ITGB3 mRNA. The more intensive knockdown of ITGB3 in I5 cells and the concomitant mildly anti-proliferative and distinctly anti-migratory effects were reason to select the I5 clone for further investigations.

### ITGB3 knockdown inhibits breast cancer skeletal metastasis

To study the effects of the conditional ITGB3 knockdown in vivo, I5 cells were examined in a nude rat model for breast cancer skeletal metastasis. Seven experimental rats received doxycycline via the drinking water, starting 3 days prior to intra-arterial tumor cell inoculation and lasting for further 2 weeks. I5 cells were injected into four rats and cells from the parent cell clone were injected into three control rats. The group sizes were chosen to be as low as they still are suited to result in a significant difference.

**Fig. 4:**
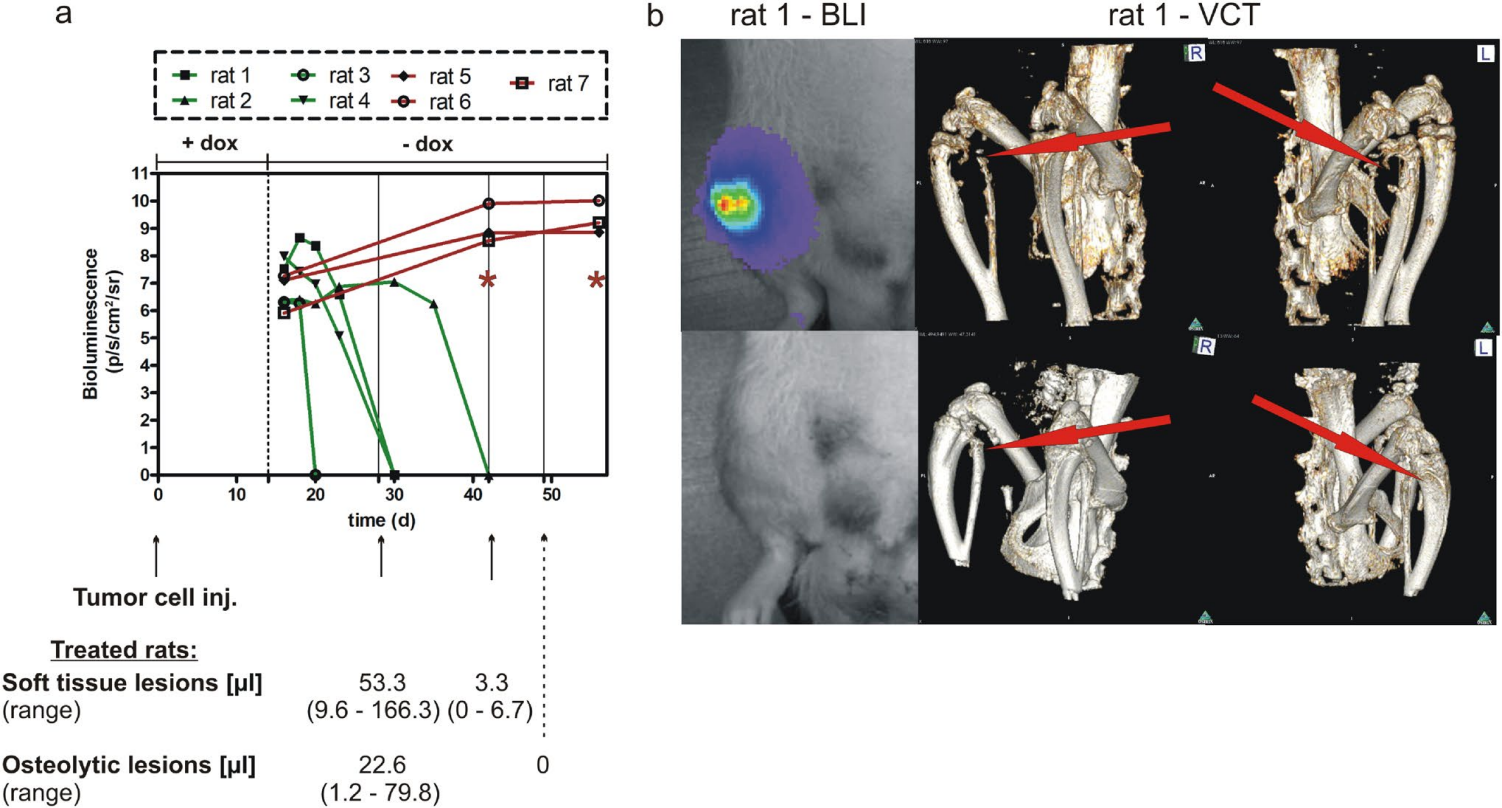
Breast cancer skeletal metastasis inhibition after ITGB3 knockdown **a** The line graph (*x*-axis: time (in days) after tumor cell injection (tumor cell inj.); *y*-axis: bioluminescence in photons/second/cm^2^/steradian) shows quantitation of bioluminescence imaging (BLI) signals from control rats (red color) and rats treated with a miRNA against integrin β3 (green color). Below the graph, a time line is given, indicating the (relative) time of tumor cell injection, and of magnetic resonance imaging (MRI; indicating the volume of soft tissue lesions) and volume computed tomography (VCT; indicating the volume of osteolytic lesions) measurements, as indicated by arrows. Below this time line, the mean values and corresponding ranges are given for the treated rats, only. The breast cancer skeletal metastases of experimental rats were monitored initially after discontinuation of the doxycycline intake (the time point is indicated with a dashed line). The cells of the specific cell clone I5 were injected into the saphenous artery of the first four rats (rat 1 to rat 4). The other three rats (rat 5 to rat 7) received cells from the parent cell clone and these rats were used as controls. The difference in light emission between miRNA treated and control rats was highly significant (*p* < 0.001) as indicated by red asterisks. **b** BLI images (left panel) and VCT images (middle and right panels) show the status of rat 1 at 20 and 28 days (upper panel) and at 30 and 49 days after tumor cell inoculation (lower panel), respectively. Clearly, the luminescence signal is no longer visible at the later time point, and the bones show re-calcification

The elaborate operation procedure for establishing this orthotropic model includes general anesthesia of the experimental rats and careful preparation of the place for injection in the right hind leg’s distal femoral artery. This is the area, where the saphenous and popliteal arteries branch off the femoral artery. After the tumor cell injection into the saphenous artery, the cells were directed into the popliteal artery, the operation site was carefully closed (for a more detailed description of the surgical procedures see Zepp et al. (2011)). This described tumor cell application restricts the growth of skeletal metastasis to a single limb, only, which contrasts with often used mouse models. For follow-up, the tumor growth (growth of soft tissue lesions) was monitored until palpable lesion sizes were detected in the animals, which was realized after a 2-week period of doxycycline intake. The subsequent discontinuation of doxycycline administration led to stimulation of the expression of luciferase and the specific miRNA targeting ITGB3 mRNA in rats inoculated with the I5 clone, whereas control rats showed only expression of luciferase. The presence of luciferase allowed to start monitoring the tumor size development by bioluminescence imaging (BLI) for measuring the effect of the miRNA mediated ITGB3 knockdown in vivo (Fig. 4a). As shown in the line graph, the initial bioluminescent signals of the established tumors corresponded to 10^6^–10^8^ p/s/cm^2^/sr. For controls, these values increased by 10 to 10-fold during the subsequent observation period. Treated rats showed some variation in their response. Rat 1 showed initially a tenfold increase in light emission, which was followed by a steady decrease in tumor size, until no tumor was detectable after 16 days of exposure to the miRNA (day 30). Rat 2 showed a long period of 3 weeks with slightly increasing light emission, until a rapid decrease in light emission started, indicating tumor remission. At 42 days after tumor inoculation, the respective light signals were no longer detectable. For rat 3, the period until complete disappearance of light signals was very short, as they were absent already at 6 days after cessation of doxycycline administration. The tumor growth of rat 4 was similar to that of rat 1, with the exception of an initial increase in light emission. Taken together, the light emission of rats treated with a miRNA against ITGB3 decreased uniformly until the detection limit, though the course of tumor regression varied. When tested by unpaired t-test and corrected for multiple comparisons using the Holm-Sidak method, the light emission of control and miRNA treated rats was significantly different at day 40 after tumor cell implantation, and any time point thereafter (*p* < 0.001). In addition to bioluminescence imaging, the soft tissue lesions of miRNA treated rats were monitored by magnetic resonance imaging (MRI) after 2 and 4 weeks. The mean volume of these lesions decreased from an initially palpable size (> 500 mm^3^, corresponding to 500 µl) to 53 µl and 3.3 µl at days 28 and 42 after tumor cell implantation. Notably, at the second time point, one of four treated rats showed no measurable lesion, corresponding to a full clinical remission. The corresponding osteolytic lesions were monitored by volume computed tomography (VCT) after 28 and 49 days after tumor cell inoculation. As for the MRI results, the initial recording gave a measurable mean (22.6 µl) of lesions from four rats, which changed to ‘zero’ after 35 days of miRNA treatment in four of four rats. An example for these complete remissions is given in the lower part of Fig. 4b, which shows an intact skeletal structure of rat 1 for this time point.

### ITGB3 in exosomes in vitro and in vivo

**Fig. 5:**
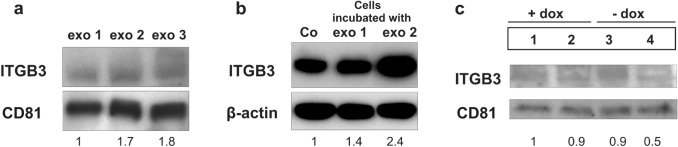
Protein levels of ITGB3 in exosomes. **a** Levels of ITGB3 in exosomes isolated from plasma of healthy rats (exo 1) and from plasma of rats with breast cancer skeletal metastasis (exo 2 and exo 3). The latter exosomes show higher levels of this protein. **b** ITGB3 in MDA-MB-231 breast cancer cells incubated with exo 1 or exo 2 for 72 h. The levels of this protein were increased after incubating the cells with exosomes isolated from plasma of rats with breast cancer lesions (exo 2). **c** ITGB3 levels in exosomes isolated in vitro from: (1) parent cell clone, cultivated in medium with doxycycline (+ dox control), (2) I5 cells cultivated in medium with dox (+ dox, specific control), (3) parent cell clone, cultivated in medium without dox (-dox control), (4) I5 cells cultivated in medium without dox (-dox, anti-ITGB3 effects). ITGB3 decreased twofold in exosomes derived from cells with conditional integrin β3 knockdown

ITGB3 was analyzed also in exosomes, which were secreted by MDA-MB-231 cells in vitro and in vivo*.* Exosomes from plasma of rats bearing MDA-MB-231 induced skeletal metastases (exo 2 and exo 3) contained about twofold higher levels of ITGB3 than exosomes from healthy rats (exo 1) (Fig. 5a). Also, MDA-MB-231 cells incubated in vitro with these exosomes derived from rat plasma (exo 2), showed 2.4-fold increased ITGB3 levels (Fig. 5b). Furthermore, exosomes secreted from the parent and I5 cells were analyzed in vitro. They were isolated from the media of these cells after cultivating them for 6 days in presence or absence of doxycycline. In comparisons to the ITGB3 levels contained in the exosomes of the three controls, this protein was twofold decreased in exosomes, secreted from the cells with conditional knockdown of ITGB3 (Fig. 5c).

### Gene modulation after conditional ITGB3 knockdown

Microarray analysis was performed after 3 or 6 days of conditional ITGB3 knockdown. From a total of 48,000 genes, only 28 protein-coding genes were modulated more than 1.5-fold in expression and showed corresponding *p* values < 0.01. All genes showing a lower fold change in expression, as observed after both time points, were disregarded. Only 11 genes were modulated after the shorter period, as seven of them were down- and four genes were upregulated. Interestingly, after the longer period (6 days) of conditional miRNA mediated ITGB3 knockdown, the number of modulated genes had increased more than twofold (to 25), with the number of downregulated genes being four times higher than that of the upregulated ones (20 versus 5).

**Fig. 6:**
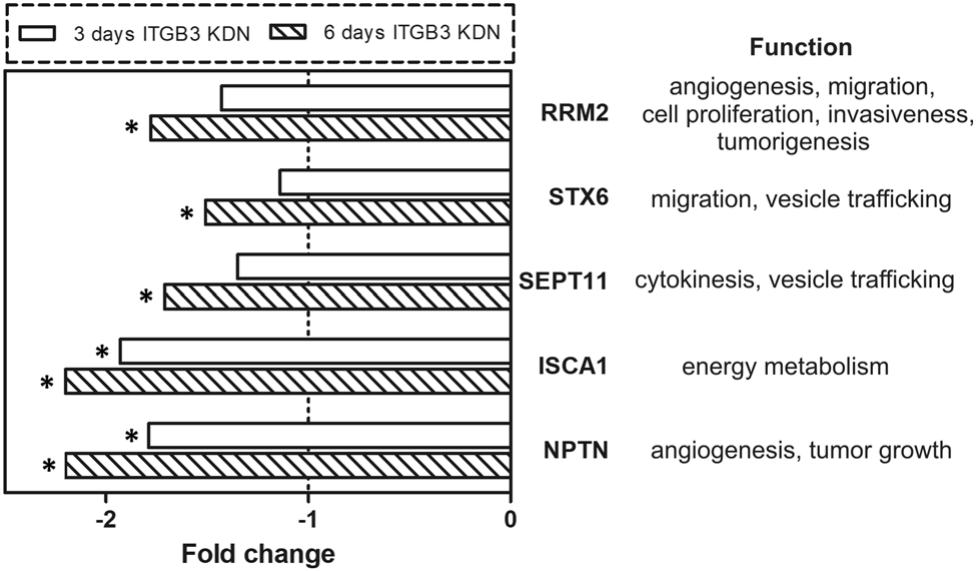
Gene modulation upon integrin beta-3 knockdown (ITGB3 KDN) Downregulation of selected genes after 3 and 6 days of ITGB3 miRNA-mediated inhibition. The gene expression of the cell clone with conditional ITGB3 knockdown was compared to that of the same cell clone cultivated in media with doxycycline (the corresponding control). The dashed line indicates a fold change without gene expression modulation, the asterisks indicate a significantly altered fold change (*p* < 0.001). The genes include NPTN (neuroplastin), ISCA1 (iron-sulfur cluster assembly 1), SEPT11 (septin 11), STX6 (Syntaxin 6), and RRM2 (ribonucleotide reductase M2 polypeptide). The right side of the figure shows the respective gene functions

The majority of modulated genes showed delayed onset or persistent downregulation (50% and 21%, respectively). The genes from the first group with ‘delayed onset’ changes were downregulated more than 1.5-fold only after 6 days, but not after 3 days of conditional ITGB3 knockdown. After the earlier time point, their modulation was less than 1.5-fold. Among these genes are *SEPT11, STX 6* and *RRM2*. The genes from the second group had persistent downregulation after both periods of ITGB3 inhibition. This group includes *NPTN* and *ISCA1*, which were downregulated by 1.9- and 1.8-fold after 3 days and 2.2-fold after 6 days of ITGB3 knockdown (Fig. 6). However, the mRNA levels of other integrin related genes with bearing for migration, like FAK and Rac1/2/3, showed no modulation.

## Discussion

For developing specific anti-metastatic therapies, deciphering the molecular mechanism of breast cancer skeletal metastasis and defining the driver genes in cancer progression is essential.

According to several published reports, integrin β3 is an interesting option, as it is expressed by breast cancer cells (Das et al. 2018) and its heterodimer αvβ3 is related to high rates of bone metastasis, tumor-associated osteolysis and colonization of the skeleton (Weilbaecher et al. 2011). Here we aimed to create a model for monitoring the anti-ITGB3 effects in breast cancer cells, starting from a freely chosen onset, and continued for a defined period in vitro or in vivo. Therefore, we created two MDA-MB-231 breast cancer cell clones with conditional doxycycline-dependent miRNA-mediated ITGB3 knockdown. These clones are able to express constitutively tTA, which in absence of doxycycline stimulates the simultaneous expression of an artificial miRNA targeting ITGB3 mRNA, and of reporter genes for firefly luciferase and red fluorescent protein mCherry. Vice versa, presence of the tetracycline derivative switches off the expression of these genes. The flow cytometry analysis of these cells revealed a stimulation of mCherry production upon removal of doxycycline, which is an indication for a concomitant stimulation of the specific artificial miRNA expression. The specificity of the respective siRNA sequence used to create the miRNA had been proven before (Reufsteck et al. 2012). Moreover, this ‘tet-off’ condition allowed monitoring the size of tumors in our nude rat model for breast cancer skeletal metastasis by bioluminescence imaging because of the simultaneous expression of firefly luciferase. The conditional miRNA-mediated ITGB3 knockdown led to a mild but significant inhibition of proliferation and a pronounced reduction in migration of the breast cancer cell clones in vitro. More importantly, partial and even complete remissions of both, soft tissue and osteolytic lesions were achieved in vivo and detected by BLI, MRI and VCT scans after 4–5 weeks of miRNA exposure.

Tumor-derived exosomes have recently been recognized as means for better understanding the mechanism of cancer progression (Hoshino et al. 2015). It is more and more clear that these exosomes could have a functional role in determining organ sites of potential metastasis, as they aid in preparing the microenvironment of the pre-metastatic niche, which supports cancer cell survival (Hoshino et al. 2015). Most probably, this property depends on the specific protein and RNA content of the tumor-derived exosomes. In this study, the exosomes isolated from plasma of rats harboring MDA-MB-231-induced skeletal metastasis showed twofold increased levels of ITGB3 compared to exosomes of control rats. Moreover, MDA-MB-231 breast cancer cells incubated with these exosomes in vitro showed an increased ITGB3 production. Correspondingly, the conditional miRNA-mediated knockdown of ITBG3 in MDA-MB-231 cells was associated with a reduced presence of this protein in their exosomes. As integrins are known as adhesion-mediating molecules (Hamidi et al. 2016), we suggest that ITGB3 plays a role in the adhesion of the tumor-derived exosomes to their target cells of the pre-metastatic niche, which is an important step that enables the horizontal transfer of other biomolecules from these exosomes to their target cells. Moreover, we hypothesize that exosomes from breast cancer cells with conditional knockdown of ITGB3 having low levels of this protein, possess a reduced capacity to modulate the pre-metastatic niche and are critical for cancer cell survival and tumor growth.

The effects observed following ITGB3 knockdown in vitro and in vivo can be explained by downregulation of specific genes, which have roles in angiogenesis (NPTN, RRM2) (Rodriguez-Pinto et al. 2009; Chen et al. 2019), tumor growth (NPTN) (Rodriguez-Pinto et al. 2009), energy metabolism (ISCA1) (Yang et al. 2019), cytokinesis (SEPT11) (Hanai et al. 2004), migration (RRM2, STX6) (Chen et al. 2019; Zhu et al. 2016), cell proliferation, invasiveness, senescence, tumorigenesis (RRM2) (Chen et al. 2019) and vesicle trafficking (SEPT11, STX6) (Hanai et al. 2004; Zhu et al. 2016). It is known that the high expression of neuroplastin (NPTN) in breast carcinomas with distant metastasis is related to a significant increase in tumor growth and angiogenesis (Rodriguez-Pinto et al. 2009). Moreover, NPTN was highly expressed in circulating tumor cells (CTCs) (Powell et al. 2012). In our study, the NPTN expression was persistently down-regulated in MDA-MB-231 breast cancer cells upon ITGB3 knockdown. Iron-sulfur cluster assembly 1 (ISCA1) is a mitochondrial protein, associated with the biogenesis of iron–sulfur clusters (Cozar-Castellano et al. 2004), which are essential cofactors for cellular processes like mitochondrial respiration and DNA metabolism (Yang et al. 2019). ISCA1 relocates Fe/S from a scaffold protein to the mitochondrial proteins from the respiratory complex, which is necessary for their stability. It has been reported that ISCA1 knockout led to impairment of the electron transport chain and energy metabolism (Yang et al. 2019). Here, following the conditional ITGB3 knockdown, ISCA1 was persistently downregulated, which might be a hint for impeded cellular energy metabolism. Ribonucleotide reductase subunit M2 (RRM2) is rate-limiting enzyme for DNA synthesis and repair, which has essential roles in cellular processes such as cell proliferation, invasiveness, migration, angiogenesis, senescence, and tumorigenesis (Chen et al. 2019). RRM2 was found to be upregulated in various breast cancer cells, especially in triple negative breast cancer (Quan et al. 2019). Worse overall survival was observed in breast cancer patients with increased RRM2, which had an enhancing effect on relapsing metastases. In line with this, it was reported that RRM2 is related to resistance of breast cancer cells to chemotherapy and endocrine agents, as suppression of RRM2 led to increased chemo-sensitivity and reversed tamoxifen-resistant cell proliferation, reduced tumor growth and cell motility, as well as activated pro-apoptotic pathways (Chen et al. 2019). In this study, we found that ITGB3 knockdown led to a progressive decrease in RRM2 levels, hence we suggest that ITGB3 might be a driver protein for this breast cancer-related protein. Septins are cytoskeletal GTPases, the function of which is related to cytokinesis and vesicle trafficking. Septin 11 (SEPT11), was reportedly co-localized with microtubules and actin stress fibers in HMEC cells (Hanai et al. 2004). Moreover, it was highly expressed in many cancers, including breast cancer (Liu et al. 2010). Here, the downregulation of SEPT11 after ITGB3 knockdown could explain the decreased migration activity of the breast cancer cells used, due to impairment of the cytokinesis/cytoskeletal dynamics. Syntaxin 6 is a vesicle transport protein, which is mainly localized in the trans-Golgi network (TGN) and to a lower degree on endosomes. It is necessary for the transport of selective cargo proteins (like oncoproteins) from the Golgi apparatus to the plasma membrane, or especially to lamellipodia, which is important for cell migration (Zhu et al. 2016). Thus, the downregulation of both SEPT11 and STX6 could provoke an impaired vesicle trafficking inside and outside of the cells (decreased formation of endosomes and exosomes). An actual decrease of the amount of secreted exosomes from breast cancer cells would reduce their efficacy in modulating the pre-metastatic niche and in subsequent organ colonization. These results are first steps in getting a mechanistic insight into the role of ITGB3, but more experiments are needed. However, the modulation of these genes allows speculating that the ITGB3 knockdown led to a decreased cellular energy metabolism, to reduced tumor growth, impaired possibilities for cytokinesis and migration, as well as to decreased vesicle trafficking (endosome and exosome formation). The suggested impaired functions are in line with the observed effects in vitro (reduced proliferation and migration) and in vivo (decreased tumor growth and complete remissions of osteolytic lesions).

In summary, the conditional inhibition of ITGB3 expression in breast cancer cells is related to anti-proliferative and anti-migratory effects in these cells as well as to decreased amounts of this protein in exosomes shed from these cells. In addition, the miRNA-mediated reduction of cellular ITGB3 synthesis in MDA-MB-231 cells is associated with partial and complete remissions of both, soft tissue and osteolytic lesions in a nude rat model for breast cancer skeletal metastasis. These effects can be explained by the specific gene modulation upon ITGB3 knockdown. Therefore, we suggest that ITGB3 has a vital function related to the progression of breast cancer cells, as this protein is instrumental for local and distal effects, mediated by direct contact or via exosomes. These findings points to integrin beta3 as valuable target for treating skeletal metastasis.
